# Long-Term Adherence to Physical Activity Among Older Veterans

**DOI:** 10.1093/geroni/igaa057.3167

**Published:** 2020-12-16

**Authors:** Candace Brown

**Affiliations:** University of North Carolina at Charlotte, Charlotte, North Carolina, United States

## Abstract

The benefits of physical activity (PA) are well-established and it is recommended that older adults achieve at least 150 to 300 minutes of moderate intensity PA and strengthening activities weekly. However, only 54.0% and 23.2% of older adults achieve these recommendations for endurance and strengthening (respectively), and 48% dropout within the first 6-months. Most PA research focuses on the 6-month initiation phase leaving a gap regarding long-term adherence. We explored predictors of long-term adherence (>2-years) to PA from 97participants at 6-month follow-up and yearly surveys. Variables examined included age, race, gender, body mass index (BMI), and self-reported comorbidities, symptoms, physical function, and barrier-specific self-efficacy scale (α-level 0.05). Lower BMI (29.1±5.1 versus 31.6±6.5, p=0.047) and higher self-efficacy to overcome environmental barriers (p=0.016) and social isolation (p=0.05) were associated with long-term adherence. Self-efficacy to overcome environmental and social barriers should be addressed to promote long-term adherence to exercise among older adults

